# Murine Nephrotoxic Nephritis as a Model of Chronic Kidney Disease

**DOI:** 10.1155/2018/8424502

**Published:** 2018-03-05

**Authors:** M. K. E. Ougaard, P. H. Kvist, H. E. Jensen, C. Hess, I. Rune, H. Søndergaard

**Affiliations:** ^1^Department of Diabetes Complications Pharmacology, Novo Nordisk, Maaloev, Denmark; ^2^Department of Veterinary and Animal Sciences, University of Copenhagen, Frederiksberg, Denmark; ^3^Department of Histology and Bioimaging, Novo Nordisk, Maaloev, Denmark

## Abstract

Using the nonaccelerated murine nephrotoxic nephritis (NTN) as a model of chronic kidney disease (CKD) could provide an easily inducible model that enables a rapid test of treatments. Originally, the NTN model was developed as an acute model of glomerulonephritis, but in this study we evaluate the model as a CKD model and compare CD1 and C57BL/6 female and male mice. CD1 mice have previously showed an increased susceptibility to CKD in other CKD models. NTN was induced by injecting nephrotoxic serum (NTS) and evaluated by CKD parameters including albuminuria, glomerular filtration rate (GFR), mesangial expansion, and renal fibrosis. Both strains showed significant albuminuria on days 2-3 which remained significant until the last time point on days 36-37 supporting dysfunctional filtration also observed by a significantly declined GFR on days 5-6, 15–17, and 34–37. Both strains showed early progressive mesangial expansion and significant renal fibrosis within three weeks suggesting CKD development. CD1 and C57BL/6 females showed a similar disease progression, but female mice seemed more susceptible to NTS compared to male mice. The presence of albuminuria, GFR decline, mesangial expansion, and fibrosis showed that the NTN model is a relevant CKD model both in C57BL/6 and in CD1 mice.

## 1. Introduction

Animal models with clinical and pathological features of human chronic kidney disease (CKD) are highly warranted to advance novel therapies for CKD and would enable a deeper understanding of the pathogenesis and thereby more target-specific therapies. CKD is defined clinically by prolonged and progressive loss of kidney function measured by a declined glomerular filtration rate (GFR) and the presence of albuminuria with pathological findings of mesangial expansion, inflammation, and renal fibrosis [1].

Prior work has documented limitations of the classical murine models of CKD including the unilateral ureteral obstruction (UUO), 5/6 nephrectomy, and diabetic nephropathy models [2, 3]. The pathogenesis of the UUO and the 5/6 nephrectomy models is difficult to study. The unobstructed kidney in the UUO model compensates for the loss of function in the obstructed kidney [2]. In the 5/6 nephrectomy model, only a small amount of kidney tissue is available, and the model requires a difficult technical surgery making it difficult to reproduce [3–6]. Models of diabetic nephropathy also have their limitations as both the classical streptozotocin- (STZ-) induced model and the db/db model develop slowly and often only show mild signs of CKD [7].

The pathogenesis in the nonaccelerated nephrotoxic nephritis (NTN) model is initiated by anti-glomerular IgGs that impair the glomerular filtration barrier and induce proteinuria and inflammation. The NTN model is largely described as a model of acute glomerulonephritis, and the knowledge of the long-term pathogenesis and strain differences in the nonaccelerated murine NTN model is therefore limited [3, 8–10]. The standard of care for CKD has for many decades consisted of treatment with angiotensin-converting-enzyme inhibitors (ACE-I's) or angiotensin receptor blockers that reduce albuminuria and slow down the disease progression [11]. A previous study in the NTN rat has demonstrated that ACE-Is reduce albuminuria and glomerular sclerosis indicating the NTN model is a suitable model of human CKD [12].

The murine C57BL/6 strain is the most commonly used genetic background of inbred strains in research. However, these strains are resistant to the development of CKD in the standard model; 5/6 nephrectomy unless hypertension is induced in addition [13]. Furthermore, the nephrectomy and the streptozotocin models show increased CKD severity in the outbred stock—CD1—compared to the C57BL/6 mice [14–16]. The influence of gender on CKD is still debated. However, the prevalence of CKD tends to be increased in women, but the CKD is more severe in men [17].

The murine model of NTN allows investigations of the immune mechanisms in rapidly progressive glomerulonephritis (GN). Few chronic experiments have evaluated the more chronic features of the induced kidney damage by testing therapeutic strategies for renal fibrosis in the accelerated NTN model with the use of immunisation and adjuvants [18, 19]. Therefore, we performed an in-depth time course study using both C57BL/6 and CD1 male and female mice in the nonaccelerated NTN model by measuring their acute and progressive chronic manifestations of CKD.

## 2. Materials and Methods

### 2.1. Experimental Animals and Study Design

C57BL/6 mice (8–10 weeks) were purchased from Taconic (Ry, Denmark) and CD1 mice (8–10 weeks) were purchased from Charles River (Germany). The mice were housed in a facility with a 12 h light/dark cycle with free access to water and Altromin chow. Before the study, the mice were acclimatised for one week. All animal experiments were approved by the Danish Animal Inspectorate and the Novo Nordisk ethical review board. Mice were euthanised if they experienced >20% weight loss or compromised health.

Prior to termination, mice were induced with isoflurane, and the kidneys were perfused with 0.9% NaCl with Heparin (10 U/ml) before isolation and collection.

**Figure 1:**
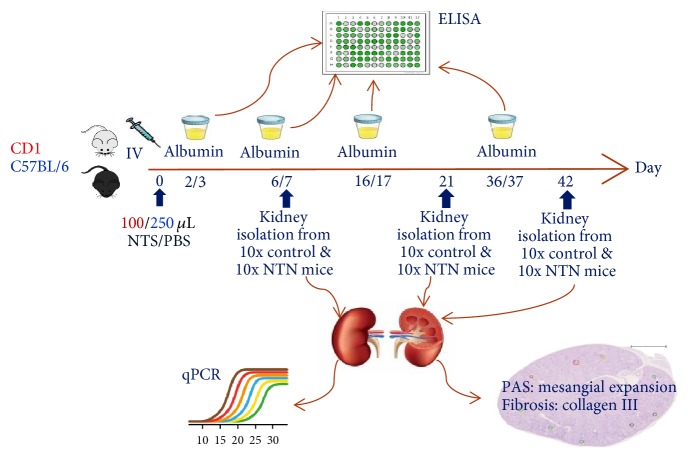
Study set up in C57BL/6 and CD1 mice.

Initial dose titration studies were conducted to determine the optimal dose of NTS in both strains using 50–250 *μ*l of NTS. The optimal doses were used in two parallel experiments conducted in C57BL/6 and CD1 mice to determine the time course of CKD disease development. Nonaccelerated NTN was induced by a single tail-vein injection of 250 *μ*l (C57BL/6) and 100 *μ*l (CD1) of sheep anti-rat NTS (Probetex, San Antonio, USA, PTX-001S lot#199-8). Control mice received PBS by same administration and volume. The study design followed a randomised block design with 30 mice in each group. At days 7, 21, and 42 ten mice per group were sacrificed, and plasma and kidneys were collected (Figure 1). The mice were weighed on day 0 just before NTS was injected and hereafter twice a week, and the percentage weight change was calculated throughout the study. The gender studies in CD1 and C57BL/6 are described in supplementary materials.

### 2.2. Urine and Plasma Analysis

Urine was collected by metabolic caging to measure the urinary albumin concentration and to calculate the urinary albumin excretion rate (UAER). Mice were single-housed in metabolic cages for 18 hours on days 2-3, 6-7, 16-17, and 36-37. Urinary markers were measured by ELISA: albumin (Bethyl Laboratories, cat.no. E90-134), Cystatin C (R&D systems, Minneapolis, MN Cat. number MSCTCO) and TNFR1 (R&D systems, Minneapolis MN Cat. number MRT10). Urinary creatinine was measured by high-performance liquid chromatography (HPLC). Creatinine was measured in serum by acetonitrile deproteinization, followed by isocratic, cation exchange HPLC as previously described [20].

Blood plasma was prepared from blood samples collected on days 7, 14, 21, 28, and 42. Serum Amyloid P (SAP) was measured by ELISA (Genway, San Diego, CA). Cystatin C was measured using ELISA (R&D systems, Minneapolis, MN Cat. number MSCTCO).

### 2.3. Glomerular Filtration Rate

The glomerular filtration rate (GFR) was measured in CD1 female mice (8–10 weeks) in an additional study with similar induction of NTN. The glomerular filtration rate (GFR) was measured on days 5-6, 15–17, and 34–37 by preclinical transdermal GFR monitors (Medibeacon GmBH, Mannheim, Germany) as previously described [21]. In short, a square of 2 × 2 centimetres fur on the back of the mice were depilated 24 hours prior to GFR measurements. A stock of FITC-sinistrin (Medibeacon GmBH, Mannheim, Germany) was prepared and stored in aliquots at −20°C. Prior to injection of FITC-sinistrin, the mice were lightly anaesthetised and the GFR monitor was adhered to the depilated area by adhesive tape. The mice were injected intravenously in the tail vein with 7.5 mg/100 g BW of FITC-sinistrin and placed in an enriched cage for one hour.

### 2.4. Kidney Gene Expression by Real-Time Quantitative PCR

Following euthanasia, one-half of the left kidney was snap frozen in liquid nitrogen and stored at −80°C. Frozen kidney tissue was homogenised in Qiazol reagent, RNA was isolated using the RNAeasy Mini kit as described by the suppliers (Qiagen, Mississauga, ON, Canada) and cDNA was generated using SuperScript VILO cDNA Synthesis kit (Life Technologies, Burlington, ON, Canada). Afterwards Real-Time Quantitative PCR (RT qPCR) was performed with Gene Expression Master Mix (Life Technologies) using a 7900HT Fast Real-Time PCR System. Specific gene expression was measured with the following Taqman assays (Life Technologies): C3 (Mm01232779_m1, C3), procollagen 3a1, (Mm01254476_m1, Col3a1), Fibronectin (Mm01256744_m1, Fn1), and PAI-1 (Mm00435858_m1, Serpine1). RT qPCR was performed in triplicate, and the values normalised to GAPDH and RPL27 as previously described [22].

### 2.5. Histological Analysis

Perfused kidneys were fixed in 4% paraformaldehyde for 30 h, processed by standard procedures through graded concentrations of alcohol and xylene and embedded in paraffin. Paraffin sections of 3 microns were stained with Periodic Acid-Schiff (PAS) and scanned using the Nanozoomer 2.0 (Hamamatsu Photonics K.K., Hamamatsu, Japan) at a magnification of ×40. The mesangial expansion was evaluated in a blinded fashion as 20 glomeruli of each kidney were assessed and graded into four categories: 0 (no mesangial expansion), 1 (mild mesangial expansion, mesangial matrix wide < 2 nucleus diameter), 2 (moderate mesangial expansion, mesangial matrix wide < 4 nucleus diameter), and 3 (severe mesangial expansion, > 4 nucleus diameter). The 20 glomeruli of each mouse were evaluated for metaplasia, segmental or global sclerosis. Furthermore, the kidneys were evaluated for the presence of protein casts. The tubular casts were visualised as solidification of protein in the lumen of the kidney tubules. The metaplasia was visualised as a change from flattened parietal epithelium to cuboidal epithelium lining the glomerulus. The segmental glomerulosclerosis was visualised as glomeruli that showed scarring of small sections of the glomeruli, while global glomerulosclerosis was visualised as totally scarred glomeruli.

### 2.6. Immunohistochemistry

Immunohistochemical (IHC) staining of collagen III was performed to quantitate renal fibrosis. Paraffin-embedded sections were deparaffinised and hydrated followed by antigen retrieval with proteinase K (10 *μ*g/ml) treatment for 10 minutes at 37°C and treated in TBS for 5 minutes. Endogenous peroxidase was blocked using 0.5% H_2_O_2_ in TBS for 20 minutes, and sections were incubated with avidin and biotin for 10 min each. Sections were incubated with TBS mixed with 7% donkey, 3% mouse serum, and 3% skimmed milk. Subsequently, sections were incubated overnight at 4°C with goat anticollagen III (Southern Biotech, Birmingham, USA) diluted in 7% donkey, 3% mouse serum, and 0.5% skimmed milk in TBS. The next day, the sections were incubated in biotinylated donkey anti-goat IgG (cat. number 705-065-147) diluted in 7% donkey, 3% mouse serum, and 0.5% skimmed milk in TBS and afterwards in vectastain ABC complex in TBS. Specific binding of antibodies was visualised by enzymatic conversion of the chromogenic substrate DAB into a brown precipitate by HRP activated by hydrogen peroxide. The slides were counterstained with haematoxylin.

### 2.7. Digital Image Analysis for Quantification of Fibrosis

All slides were scanned using the Nanozoomer 2.0 at an original magnification of ×40. The image analysis was performed using Visiopharm Integrator System software (VIS; Visiopharm, Hoersholm, Denmark). An automated tissue detection protocol was performed as previously published [23]. Evaluation of the collagen III staining was determined in a region of interest (ROI) restricted to the cortex region. Within the ROI a threshold (∞–70) analysis was performed using HDAB-DAB channel as previously published [23].

### 2.8. Statistics

Statistical analyses were performed using GraphPad Prism (v6.5; GraphPad Software, CA), and data were presented as the mean ± standard deviation (SD). D'Agistino-Pearson normality test was performed. Normally distributed data were analysed by one-way ANOVA multiple testing with Turkey's correction, and nonnormally distributed data were analysed using Kruskal-Wallis multiple testing with Dunn's correction. Two-way ANOVA was applied for comparing CD1 and C57BL/6 mice at different time points. A *P* value < 0.05 was accepted as statistically significant.

## 3. Results

### 3.1. CD1 Mice Display Increased Susceptibility to NTS Compared to C57BL/6 Mice

**Figure 2:**
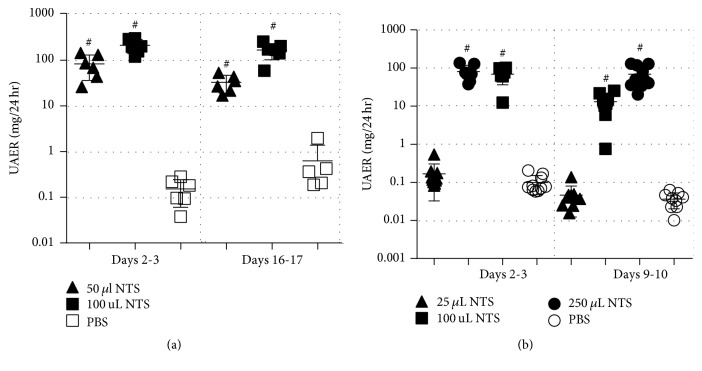
*CD1 mice are more susceptible to NTS measured by albuminuria compared to C57BL/6 mice*. (a) Scatter plot showing the 24 h urinary albumin excretion rate (UAER) of CD1 mice over time. (b) Scatter plot showing the 24 h urinary albumin excretion rate (UAER) of C57BL/6 mice over time. Data are shown as mean ± SD. ^#^*P* < 0.0001 NTS groups (*n* = 5-6) versus PBS group (*n* = 5-6) by one-way ANOVA.

The initial dose titration studies showed that mice subjected to NTS developed albuminuria in a dose-dependent manner in both CD1 mice (Figure 2(a)) and C57BL/6 mice (Figure 2(b)). C57BL/6 mice subjected to 250 *μ*l NTS showed an increased urinary albumin excretion rate (UAER) on days 9/10 compared to the groups subjected to 25 or 100 *μ*l NTS (Figure 2(b)). The dose of 250 *μ*l NTS severely affected the welfare of the CD1 mice, and 65% of this group were euthanised shortly after study initiation due to severe weight loss and signs of compromised health. A gross pathological evaluation showed that these mice had severe glomerulonephritis, and consequently, this group was excluded. CD1 mice subjected to 100 *μ*l NTS showed an increased UAER on days 16/17 compared to the group subjected to 50 *μ*l NTS (Figure 2(a)).

Based on these results, 250 *μ*l and 100 *μ*l NTS were selected for subsequent experiments in C57BL/6 and CD1, respectively.

### 3.2. NTS Induces Albuminuria, GFR Decline, and Transient Weight Loss

**Figure 3:**
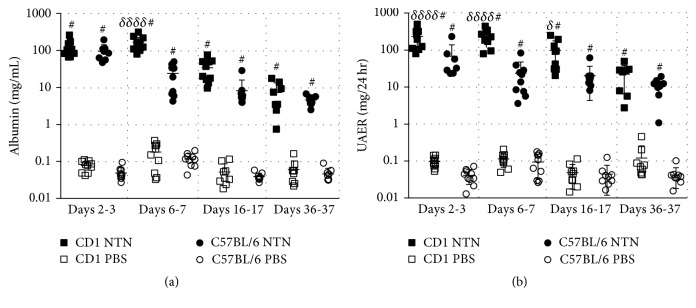
*NTS induces significant and chronic increase in UAER and urine albumin concentration*. (a) Scatter plot showing the 24 h urinary albumin excretion rate (UAER) on days 2-3, 6-7, 16-17, and 36-37. (b) Scatter plot showing urinary albumin concentration on days 2-3, 6-7, 16-17, and 36-37. Data are shown as mean ± SD. ^#^*P* < 0.0001 NTN groups versus PBS groups and ^*δ*^*P* < 0.05, ^*δδδδ*^*P* < 0.0001 CD1 NTN versus B6 NTN groups by two-way ANOVA (*n* = 10).

**Figure 4:**
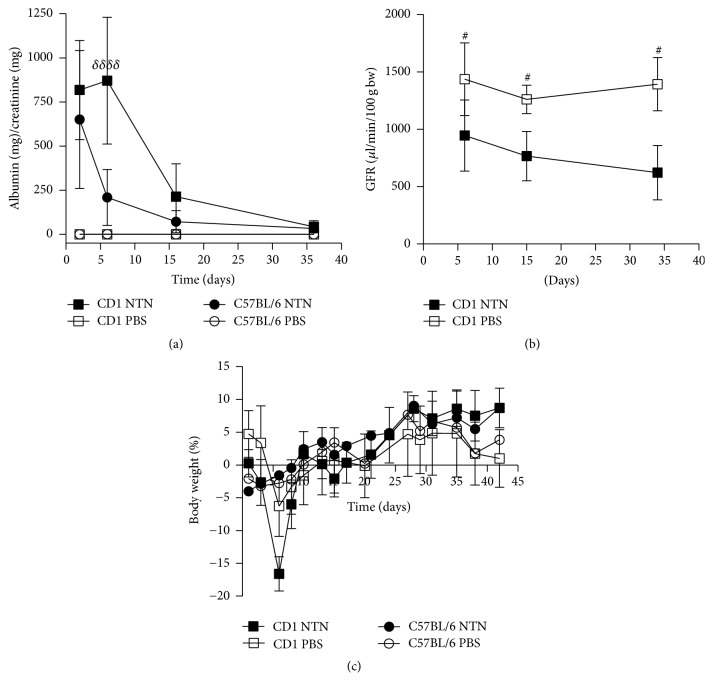
*NTS induces albuminuria, GFR decline, and transient weight loss*. (a) Graph showing the average urinary albumin/creatine ratio (mg/mg) on days 2-3, 6-7, 16-17, and 36-37. (b) Graph showing the mean glomerular filtration rate (*μ*l/min/100 g body weight (bw)) in CD1 mice on days 5-6, 15–17, and 34–37. (c) Graph showing the average percentage change in body weight over time. Data are shown as mean ± SD. ^#^*P* < 0.05 CD1 NTN versus CD1 PBS by one-way ANOVA (*n* = 10); ^*δδδδ*^*P* < 0.0001 CD1 NTN versus B6 NTN groups by two-way ANOVA (*n* = 10).

NTN induction in both strains resulted in significantly increased UAER compared to the healthy controls on days 2-3, 6-7, 16-17, and 36-37 after NTS injection (Figure 3(a)). Furthermore, the albumin concentrations of the NTN urine samples were significantly increased compared to the control urine samples at all-time points (Figure 3(b)). The CD1 NTN mice developed significantly increased UAER on days 2-3, 6-7, and 16-17 compared to C57BL/6 NTN mice (Figure 3(a)). No difference in UAER was observed between C57BL/6 male and female mice, but the female NTN mice showed a trend towards increased UAER (*P* = 0.0708 at 6 weeks and *P* = 0.0746 at 10 weeks, data not shown). However, CD1 NTN female mice developed significantly increased UAER compared to male NTN mice on days 35-36 (Supplementary material, Figure 1). The urinary albumin creatinine ratio (UACR) showed similar temporal dynamics as the UAER. The UACR peaked on days 6-7 and 2-3 in CD1 and C57BL/6 NTN mice, respectively (Figure 4(a)). The NTN induction caused a significant decrease in GFR on days 5-6, 15–17, and 34–37 in CD NTN mice compared to their controls. NTS induced transient body weight loss from day one until day 6; C57BL/6 NTN mice showed significantly greater weight loss (mean: −16,9%) compared to the CD1 NTN mice (mean: −6,3%). However, both CD1 and C57BL/6 NTN mice recovered to their initial body weight within 20 days and gained weight throughout the study (Figure 4(c)).

### 3.3. NTS Induces Significant Urinary Excretion of Cystatin C and Tumour Necrosis Factor Receptor 1

**Figure 5:**
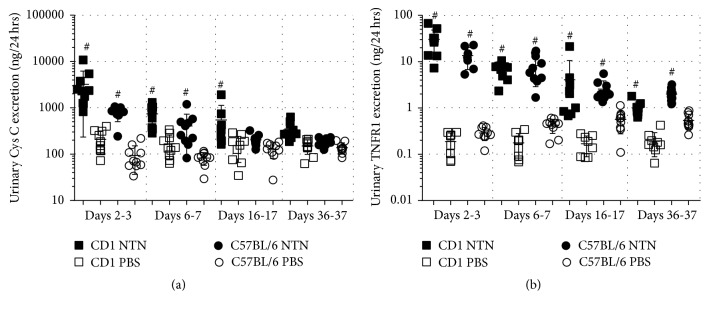
*NTS induces significant urinary excretion of Cystatin C and TNFR1*. (a) Scatter plot showing the 24 h urinary Cystatin C excretion rate over time measured by ELISA. (b) Scatter plot showing 24 h urinary TNFR1 excretion rate over time measured by ELISA. Data are shown as mean ± SD. ^#^*P* < 0.01 NTN groups versus PBS groups by two-way ANOVA (*n* = 10).

The urine analysis showed that the 24-hour urinary Cystatin C excretion was significantly increased on days 2-3, 6-7, and 16-17 in CD1 NTN mice and on days 2-3 and 6-7 in C57BL/6 NTN mice compared to control mice (Figure 5(a)). The 24-hour urinary excretion of tumour necrosis factor receptor 1 (TNFR1) was also significantly increased on days 2-3, 6-7, 16-17, and 36-37 in both strains (Figure 5(b)).

### 3.4. NTS Induces Systemic Inflammation and Elevation of a GFR Marker

**Figure 6:**
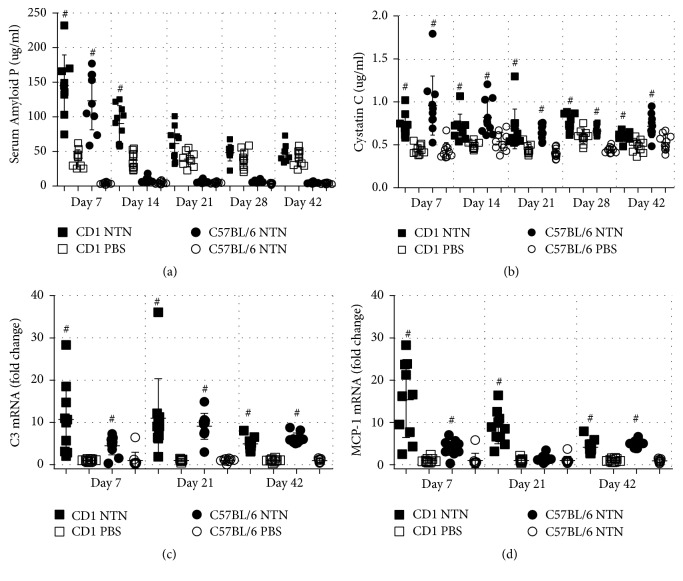
*NTS induces systemic inflammation and elevation of GFR marker*. (a) Scatter plot showing the SAP plasma concentration on days 7, 14, 21, 28, and 42 measured by ELISA. (b) Scatter plot of Cystatin C plasma concentration days 7, 14, 21, 28, and 42 measured by ELISA. (c) Scatter plot showing mRNA expression in whole kidney tissue of C3 and MCP-1 (CCL2) on days 7, 21, and 42, expressed as fold change. Data are shown as mean ± SD. ^#^*P* < 0.001 NTN groups versus PBS groups by two-way ANOVA (*n* = 10).

The plasma levels, of the acute phase protein SAP, were significantly increased on day 7 in C57BL/6 mice and days 7 and 14 in CD1 mice injected with NTS (Figure 6(a)). Supporting this notion, Cystatin C, a marker of inflammation and GFR, was also significantly increased on days 7 and 14 in both C57BL/6 and CD1 following NTS. In addition, Cystatin C was also significantly increased on days 21, 28, and 42 in both strains compared to control mice (Figure 6(b)). The inflammatory response was further investigated by complement involvement by looking at mRNA levels of C3. NTN induction significantly increased C3 gene expression in both strains on days 7, 21, and 42 (Figure 6(c)).

### 3.5. NTS Induces Chronic Kidney Injury with Mesangial Expansion and Renal Fibrosis

**Figure 7:**
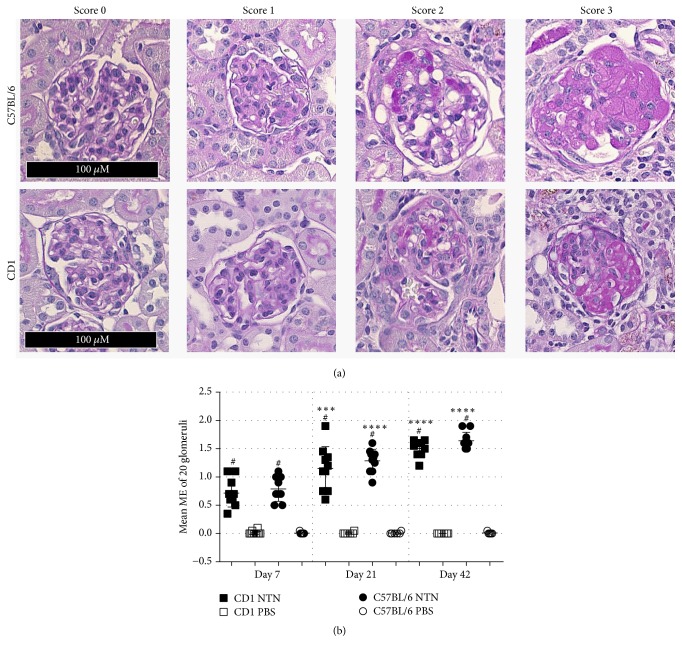
*NTS causes chronic and progressive glomerular mesangial expansion in C57BL/6 and CD1 mice*. (a) Representative glomeruli showing scores 0, 1, 2, and 3 of mesangial expansion. (b) Scatter plot showing the mean glomerular mesangial expansion (ME) score on days 7, 21, and 42. Data are shown as mean ± SD. ^#^*P* < 0.0001 NTN groups versus PBS groups; ^*∗∗∗*^*P* < 0.001 NTN group day 7 versus NTN group day 21; ^*∗∗∗∗*^*P* < 0.0001 NTN group day 21 versus NTN group day 42 by two-way ANOVA and using Kruskal-Wallis multiple testing (*n* = 10).

The mesangial expansion was observed in both CD1 and C57BL/6 NTN mice on days 7, 21, and 42 compared to their healthy controls (Figures 7(a) and 7(b)). The glomerular mesangial expansion progressed over time as it significantly increased from days 7 to 21 and from days 21 to 42 in both strains (Figure 7(b)). Both strains showed 20–22% segmentally sclerosed glomeruli on days 7, 21, and 42 and developed increased globally sclerosed glomeruli over time (2% on day 7 and 13% on day 42 (Table 1)). In addition, both strains developed metaplasia in 24–29% of the assessed glomeruli on days 7, 21, and 42. Tubular casts within cortex were present at all-time points (Table 1).

Moreover, hypercellular glomeruli, tubular proliferation, and dilatation were observed together with increasing immune cells accumulating in the tubulointerstitium and infiltrating the periglomerular space surrounding glomeruli.

**Figure 8:**
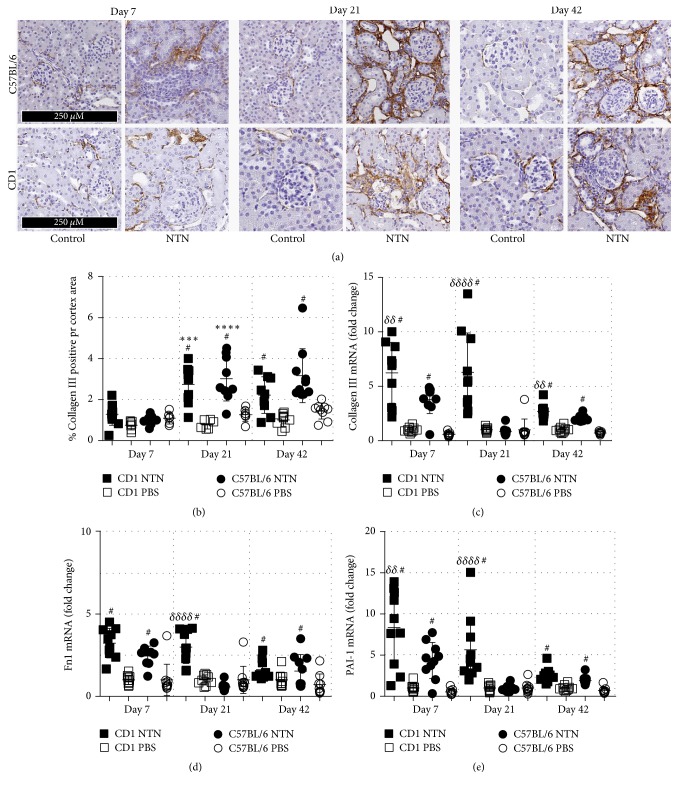
*NTS induces chronic renal fibrosis*. (a) Representative images of tubulointerstitial fibrotic area visualised by immunohistochemical collagen III staining. (b) Semiquantification of collagen III positive area of the cortex area. (c) Scatter plot showing mRNA expression in whole kidney tissue of Col3a1 as fold change. (d) Fibronectin (Fn1) mRNA expression as fold change. (e) PAI-1 (Serpine-1) mRNA expression as fold change. Data are shown as mean ± SD. ^#^*P* < 0.0001 NTN groups versus PBS groups; ^*∗∗∗*^*P* < 0.001 NTN day 7 versus NTN day 21; ^*∗∗∗∗*^*P* < 0.0001 NTN day 7 versus NTN day 21; and ^*δδ*^*P* < 0.01, ^*δδδδ*^*P* < 0.0001 CD1 NTN versus B6 NTN groups by two-way ANOVA (*n* = 10).

NTS induced similar significantly increased collagen III accumulation, and thereby renal fibrosis was observed already on day 21 in both strains (Figures 8(a) and 8(b)) compared to healthy controls. Renal fibrosis remained significantly increased on day 42 compared to controls, but it did not progress from day 21 (Figure 8(b)). No difference in collagen III accumulation was observed between C57BL/6 male and female NTN mice. However, only the female C57BL/6 NTN mice developed significantly increased collagen III deposition compared to their healthy controls (Supplementary Material, Figure 2). The female NTN mice developed significantly increased collagen III deposition compared to male CD1 NTN mice (Supplementary Material, Figure 1).

To further investigate the development of kidney fibrosis and matrix remodeling, mRNA levels of collagen type III (col III), fibronectin (fn1), and PAI-1 (Serpine1) were quantified. The NTN induction significantly increased the mRNA levels of collagen type III and PAI-1 in CD1 mice on days 7, 21, and 42 and in C57BL/6 mice on days 7 and 42 (Figures 8(c)–8(e)). The fibronectin mRNA levels were significantly increased in CD1 NTN mice on days 7 and 21 and in C57BL/6 mice on day 7 compared to control mice. In general, the CD1 female NTN mice showed increased mRNA levels on the profibrotic genes compared to C57BL/6 NTN mice, and in addition, C57BL/6 female NTN mice developed significantly increased collagen III mRNA levels compared to C57BL/6 male NTN mice (Supplementary Material, Figure 2).

## 4. Discussion

The nonaccelerated NTN model is a widely used model of acute GN, but no characterisation of the chronic progression of the disease is thoroughly described in mice. The nonaccelerated NTN model has previously been characterised in the rat, where NTN induces an autologous (acute) phase characterised by inflammation and severe proteinuria and a heterogeneous (chronic) phase characterised by glomerular lesions [24–26]. In this study we show that NTS injection in both CD1 and C57BL/6 mice induced not only an acute phase, as previously described, but also several hallmarks of CKD including albuminuria, GFR decline, mesangial expansion, inflammation, and renal fibrosis which were significantly present in the later stages of the induced NTS kidney damage.

The NTS induction significantly increased albuminuria, already on days 2-3 in both CD1 and C57BL/6 mice, suggesting an acute leakage of protein as soon as anti-GBM antibodies are deposited. The mean albuminuria declined around days 16-17, but significant elevation in UAER and urine albumin concentration remained at days 36-37 in both strains at approximately 2 logs above controls. At days 36-37 the ACR were still in the range of 33–43 mg/mg which is above the observed ACR of CKD models such as renal ablation models, UUO, and the STZ model supporting that the NTN model is a potential CKD model [27–29].

Decreased GFR is a hallmark of CKD and GFR is estimated or measured in patients for confirming diagnosis [30]. Thus, the observed GFR decline on days 5-6, 15–17, and 34–37 demonstrates that the NTN model resembles features of human CKD. The significantly increased urinary albumin concentration, UACR, UAER, and the GFR decline point towards glomerular impairment and thereby kidney dysfunction in the NTN mice [31]. Furthermore, the significant urinary excretion of Cystatin C indicates that NTN induction causes tubular dysfunction. Cystatin C is freely filtered by the glomerulus and is in healthy individuals almost 100% reabsorbed by the tubules and catabolised. On days 36-37 the urinary Cystatin C excretion in the NTN mice had returned to baseline levels which might be explained by increased reabsorption of the remaining functional nephrons or a resolution of the tubules.

The increased urinary excretion of TNFR1 might be explained by the phenomenon of shedding the receptors from membranes of TNF-*α* activated glomerular and tubular cells by a proteolytic process where the TNF-alpha converting enzyme (TACE) cleaves TNFR1 as an immunological response. On the other hand, Bemelmans et al. suggested that a continuous release of soluble TNFR1 occurs and the kidney clears it in healthy individuals [32]. The consistent levels of urinary TNFR1 excretion in the control mice support the hypothesis of a continuous release of TNFR1, and the significantly increased UAER at all-time points indicates a dysfunctional filtration barrier as both TNFR1 and albumin in healthy individuals are blocked by the glomerular filtration barrier by size and charge selectivity [33–35].

The pathological characterisation of human CKD is defined as the presence of kidney damage that progresses or remains over time and includes, in general, glomerular lesions and renal fibrosis [36–38]. The NTN model shows the presence of chronically progressing kidney damage detected by significant impairment of mesangial expansion and increased globally sclerosed glomeruli between each time point in both strains. Furthermore, the significant mesangial expansion on day 7 indicates a fast disease development. The progressive mesangial expansion developing on day 42 shows that the NTS causes features of chronic and progressive disease development.

In human CKD, renal fibrosis is characterised by the deposition of extracellular matrix (ECM) components including collagen III and fibronectin [39]. During pathological conditions, PAI-1 contributes to the accumulation of ECM components as PAI-1 inhibits degradation of ECM proteins [39]. NTS induced significant renal fibrosis detected by collagen III deposition on day 21 and day 42 in both strains. However, in contrast to the mesangial expansion, the renal fibrosis did not progress from day 21, suggesting that the fibrotic response observed here could be linked to the resolution of the initial inflammatory reaction in the NTN model. Interestingly, the development of renal fibrosis was somewhat similar in CD1 and C57BL/6 mice, which contradicts previous studies describing CD1 mice with an increased susceptibility to renal fibrosis in the 5/6 nephrectomy and STZ models [13, 15, 16]. However, differences in model duration and insult could be the explanation.

The significantly increased mRNA levels of collagen III, fibronectin, and PAI-1 in both strains were reduced on day 42 compared to day 7 suggesting a continued but slowed profibrotic activity which correlates with the discontinued progression of collagen III depositions from day 21.

Immune system activation and inflammation play a central role in the pathogenesis of acute kidney injury and CKD [40]. Following NTS injection both CD1 and C57BL/6 mice developed an acute phase inflammatory response evaluated by significantly elevated SAP in plasma on days 7–14. The baseline SAP levels of CD1 mice were significantly higher compared to C57BL/6 mice, which is consistent with literature describing strain differences in baseline SAP levels [41]. Cystatin C is described as being elevated during systemic inflammation [42], which could account for the elevation observed on days 7 and 14 consistently with the elevated plasma SAP. However, Cystatin C is not only increased due to inflammation as several studies describe Cystatin C as a superior marker of GFR [43, 44]. The observed decline in GFR on days 5-6, 15–17, and 34–37 in CD1 NTN mice strongly suggests that the increase in Cystatin C levels observed on days 7 and 14 is caused both by inflammation and by a decline in renal function. It is very likely that the elevated Cystatin C after day 14 is mainly a response to the declined GFR.

Activation of the complement system by immune complexes is known to occur in immune-mediated CKDs [45]. Complement 3 is synthesised by glomerular cells, and tubular cells and the local synthesised C3 has been demonstrated to play a role in the development of kidney disease [45, 46]. The upregulation of C3 mRNA levels in the NTN mice suggests that the injected anti-GBM antibodies activate the complement system which is in agreement with literature describing this phenomenon in the NTN model [47].

NTS induced a transient weight loss in both the CD1 and C57BL/6 NTN mice. The weight loss could be a response to NTS induced illness causing decreased diet and fluid intake. However, the recovered body weight after around day 10 indicates that the NTS doses are tolerated.

Comparing the nonaccelerated NTN model to other CKD models, the NTN model displays a technically easy and consistently inducible model with rapid disease progression when the optimal NTS dose is identified. We have shown that NTN mice develop a chronic stage of kidney disease within 21 days as seen by glomerulosclerosis, fibrosis, inflammation, tubular damage, elevated systemic markers of kidney damage, and albuminuria, whereas, for example, classical diabetic nephropathy models develop mild signs of CKD within 15–18 weeks [7]. The NTN model displays morphological aspects as mesangial expansion as well as inflammatory and fibrotic responses. In contrast, the widely used UUO model which also develops rapid inflammation and renal fibrosis is limited by the unobstructed kidney compensating for the obstructed kidney making it impractical to study the pathogenesis [2]. The 5/6 nephrectomy model develops glomeruli sclerosis and renal fibrosis within 12 weeks, but it requires a technically difficult surgery making it difficult to reproduce [6, 48].

The inbred C57BL/6 and outbred CD1 mice showed similar kidney disease progression. However, the CD1 mice had significantly increased UAER and mRNA levels of profibrotic genes at several time points compared to C57BL/6 mice. In addition, the strains showed different susceptibility to NTS as the doses needed to induce similar kidney damage were 100 and 250 *μ*l NTS, respectively. At present this difference is not well understood but is possibly related to their different genetic background, which could result in different binding properties of anti-GBM antibodies, different inflammatory response to antibody deposition, or different reactivity to other sheep serum components in the two strains. The C57Bl/6 mice showed only mild gender differences based on the significantly increased collagen III mRNA levels observed in NTN females compared to NTN males in week 10. Conversely, the CD1 NTN females showed significant UAER on days 35-36 and significantly increased collagen III deposition compared to NTN males indicating that the CD1 females are more susceptible to NTS compared to CD1 males.

In conclusion, we have shown that the nonaccelerated NTN model in addition to acute inflammatory kidney disease develops several chronic hallmarks of CKD such as albuminuria, GFR decline, progressive mesangial expansion, and renal fibrosis. C57BL/6 and CD1 mice showed similar disease manifestations making them both applicable to studies of the acute and chronic phases of kidney disease using the nonaccelerated NTN model. The CD1 mice did not display increased susceptibility to develop renal fibrosis as described in other CKD models. However, the CD1 mice, especially the CD1 female mice, did show a higher susceptibility to NTS which would possibly make them the more practical choice. The nonaccelerated NTN model quickly resembles hallmarks of acute and chronic CKD and its robustness and relatively simple NTS induction phase make it a valid alternative compared to other cumbersome models of CKD.

## Figures and Tables

**Table 1:** *Histopathological lesions by mouse strains*. The tubular casts were visualised as solidification of protein in the lumen of the kidney tubules. The metaplasia was visualised as a change from flattened parietal epithelium to cuboidal epithelium lining the glomerulus. The segmental glomerulosclerosis was visualised as glomeruli that showed scarring of small sections of the glomeruli, while global glomerulosclerosis was visualised as totally scarred glomeruli.

Pathologic changes NTN mice	CD1 day 7 *n* = 9	%	B6 day 7 *n* = 6	%	CD1 day 21 *n* = 10	%	B6 day 21 *n* = 6	%	CD1 day 42 *n* = 8	%	B6 day 42 *n* = 7	%
*Tubular casts (cortex)*												
Present (yes/no)	9	100	6	100	9	90	6	100	7	86	6	86

*Metaplasia*												
	47	26	35	29	54	27	32	27	38	24	33	24

*Glomerulosclerosis (segmental)*												
	39	22	24	20	44	22	25	21	35	22	20	14

*Glomerulosclerosis (global)*												
	4	2	2	2	22	11	8	7	20	13	18	13

## Supplementary Materials

Supplementary Materials Figure 1: CD1 gender differences in UAER and collagen III deposition. Figure 2: C57BL/6 gender differences in collagen III deposition and mRNA levels.
