# Supplementary material for: Murine Nephrotoxic Nephritis as a Model of Chronic Kidney Disease
Source: Int J Nephrol. 2018 Mar 5;2018:8424502. doi: 10.1155/2018/8424502 (PMC5859794; doi:10.1155/2018/8424502)
Supplement: Supplementary Materials — Figure 1: CD1 gender differences in UAER and collagen III deposition. Figure 2: C57BL/6 gender differences in collagen III deposition and mRNA levels. [file 8424502.f1.pdf]

Supplementary material

Experimental set up

A gender pilot study in CD 1 mice was conducted by injecting 8 male mice with 50 or 100 µl NTS or 100 µl PBS and 8 female mice with 100 µl NTS or PBS. The UAER was measured on days 7-8, and 35-36. The study was terminated on day 42 and the kidneys were collected for collagen III IHC.

The gender study in C57BL/6 mice was performed by inducing accelerated NTN by injecting 100 µg sheep IgG (5 mg/ml, Bethyl) in CFA on day -4. The mice were injected intravenously with 150 µl NTS on day 0 (PTX-001, lot: 362-3). The UAER was measured on days 16-17, and 36-37. Mice were sacrificed on day 42, and day 71 and the kidneys were collected for collagen III IHC and collagen III qPCR.

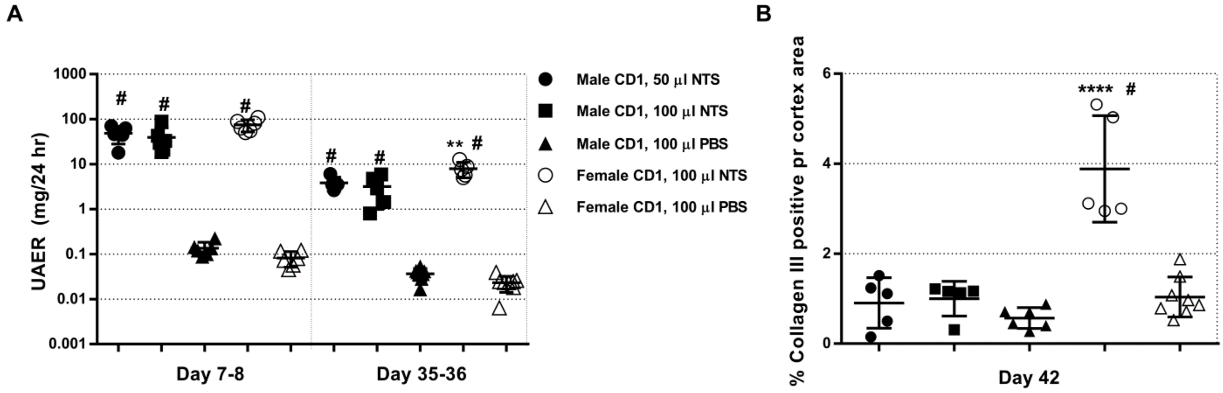

Figure 1: CD1 gender differences in UAER and collagen III deposition

(A) Scatter plot showing the 24h urinary albumin excretion rate (UAER) over time.
(B) Scatter plot showing semi-quantification of collagen III positive area of the cortex area
Data are shown as mean±SD. #, P<0.05 NTN groups (n=5) vs. PBS groups (n=6-8) and **, P<0.01, ****, P<0.0001 CD1 NTN female vs. CD1 male NTN group by one-way ANOVA.

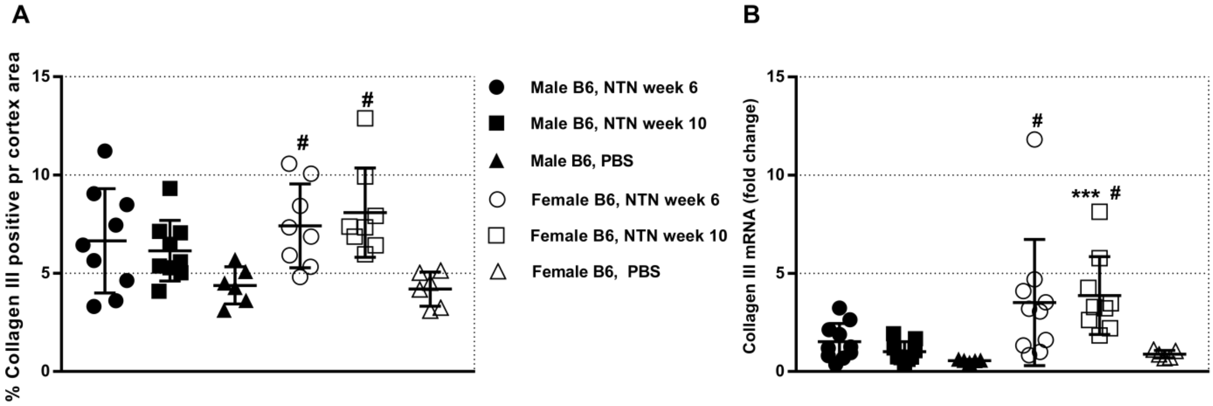

Figure 2: C57BL/6 gender differences in collagen III deposition and mRNA levels.

(A) Scatter plot showing semi-quantification of collagen III positive area of the cortex area.
(B) Scatter plot showing mRNA expression in whole kidney tissue of Col3a1 as fold change.
Data are shown as mean±SD. #, $P<0.05$ NTN groups (n=10) vs. PBS groups (n=6) and ***, $P<0.001$ CD1 NTN female vs. CD1 male NTN group by two-way ANOVA.
